# Case report: The histopathological analyses of two myelin oligodendrocyte glycoprotein antibody-associated diseases with a distinctive linear radiating gadolinium enhancement on MRI

**DOI:** 10.3389/fimmu.2024.1426236

**Published:** 2024-08-29

**Authors:** Mikito Shimizu, Goichi Beck, Shigeo Murayama, Taku Hoshi, Hiroyuki Sumikura, Kyoko Higashida, Isao Fukasaka, Yuki Shimada, Nozomi Nagashima, Tomohiro Fujioka, Naoki Hatayama, Tatsusada Okuno, Hideki Mochizuki, Manabu Sakaguchi

**Affiliations:** ^1^ Department of Neurology, Osaka General Medical Center, Osaka, Japan; ^2^ Department of Neurology, Osaka University Graduate School of Medicine, Osaka, Japan; ^3^ Department of Neurology and Neuropathology (Brain Bank for Aging Research), Tokyo Metropolitan Geriatric Hospital and Institute of Gerontology, Tokyo, Japan; ^4^ Brain Bank for Neurodevelopmental, Neurological and Psychiatric Disorders, Molecular Research Center for Children’s Mental Development, United Graduate School of Child Development, Osaka University, Osaka, Japan

**Keywords:** myelin oligodendrocyte glycoprotein antibody-associated disease, the radial linear periventricular enhancement pattern, histopathology, MRI, axonal damage, brain atrophy

## Abstract

Myelin oligodendrocyte glycoprotein antibody-associated disease (MOGAD) has highly heterogeneous clinical presentations, in which encephalitis is an important phenotype. Moreover, MOGAD has been reported to exhibit diverse imaging findings. However, there have been no previous reports of cases with perivascular radial gadolinium enhancement in periventricular regions, commonly reported in autoimmune glial fibrillary acidic protein (GFAP) astrocytopathy. In this paper, we present two cases of MOGAD with this MRI feature, both of which underwent brain biopsy for the lesions. Brain biopsies revealed perivenous demyelination and inflammation consistent with acute disseminated encephalomyelitis (ADEM), with pronounced axonal damage in Case 1 and minimal axonal involvement in Case 2. Case 1 exhibited more severe cerebral atrophy than Case 2, correlating with the extent of axonal damage. Through these cases, we highlight the heterogeneity of radiological manifestations of MOGAD, expanding the spectrum beyond previously defined MRI patterns. Furthermore, histopathological analysis revealed distinct axonal involvement as a potential prognostic marker of brain atrophy. These observations emphasize the importance of considering MOGAD in the differential diagnosis, even in cases with atypical imaging findings, and highlight the significance of brain biopsy in guiding both diagnosis and prognosis.

## 1 Introduction

Myelin oligodendrocyte glycoprotein (MOG) is a surface protein found on the outer layer of the myelin sheath and oligodendrocyte cell surfaces. MOG antibody-associated disease (MOGAD) is a relatively new type of inflammatory demyelinating disease (1, 2). MOGAD has a diverse clinical presentation, including cerebral cortical encephalitis, brainstem and cerebellar demyelinating lesions, tumefactive brain lesions, cranial neuropathies, and acute disseminated encephalomyelitis (ADEM) (2).

Recent studies have shown various brain radiological findings associated with MOGAD (3, 4). A prior report suggested that brain magnetic resonance imaging (MRI) patterns of MOGAD with encephalitis were categorized into four phenotypes: (I) multifocal hazy/poorly marginated lesions, involving both gray matter and white matter and typically involving the middle cerebellar peduncles; (II) extensive and periventricular white matter lesions resembling a “leukodystrophy-like” pattern; (III) cortical encephalitis with leptomeningeal enhancement/brain atrophy; and (IV) tumefactive demyelinating lesions (TDLs) (4). However, there are no reports of cases with perivascular radial gadolinium enhancement in periventricular regions, commonly reported in patients with autoimmune glial fibrillary acidic protein (GFAP) astrocytopathy (5).

In this report, we present two cases of MOGAD with this lesion detected on MRI scans. In addition, we report that brain biopsies, obtained from both cases, showed characteristic findings consistent with ADEM, manifesting as perivenous demyelination and inflammation. Axonal damage was observed in one case, correlating with severe brain atrophy. This study aimed to describe the radiological and histopathological features of MOGAD and contribute to a deeper understanding of its pathology.

## 2 Case 1

A 27-year-old man with no significant medical history presented with severe fatigue and fever. Approximately 3 weeks later, abnormal behavior and memory impairment, such as forgetting the route to work and missing appointments, were observed. Additionally, there was a noticeable decrease in activity levels, and the patient stopped going out, leading to hospitalization at a previous medical facility. An MRI revealed extensive white matter lesions with linear-radiating gadolinium enhancement around the ventricles. Cerebrospinal fluid (CSF) analysis revealed an elevated cell count. A brain biopsy was performed on the left frontal lobe. The patient was transferred to our hospital 2 months after symptom onset.

The routine blood test results were negative. Antinuclear antibodies (ANAs), antineutrophil cytoplasmic antibodies (ANCAs), anti-thyroid-stimulating hormone receptor (TSH) antibodies, and anti-thyroglobulin (Tg) antibodies were negative. The CSF showed no abnormal changes in nucleated cells (24/µL), protein (26 mg/dL), or glucose (76 mg/dL). Oligoclonal bands (OCBs) were detected and the IgG index was elevated (0.88). Anti-MOG and anti-NH2 terminal alpha-enolase (NAE) antibodies were positive in the serum. Anti-MOG and NAE antibodies were measured by qualitative cell-based assay and Western blot, respectively. Other autoimmune encephalitis (AE)-related antibodies against GFAP and paraneoplastic neurological syndrome (PNS)-related antibodies, including anti-Hu, anti-Ri, anti-Yo, anti-Ma2, anti-CV2, and anti-amphiphysin, were absent from the CSF and serum, respectively.

The electroencephalography (EEG) showed irregular slow waves with medium to high amplitudes in the right temporal lobe and no epileptic activity. Moreover, the chest and abdominal computed tomography (CT) showed no signs of a solid tumor.

**Figure 1:**
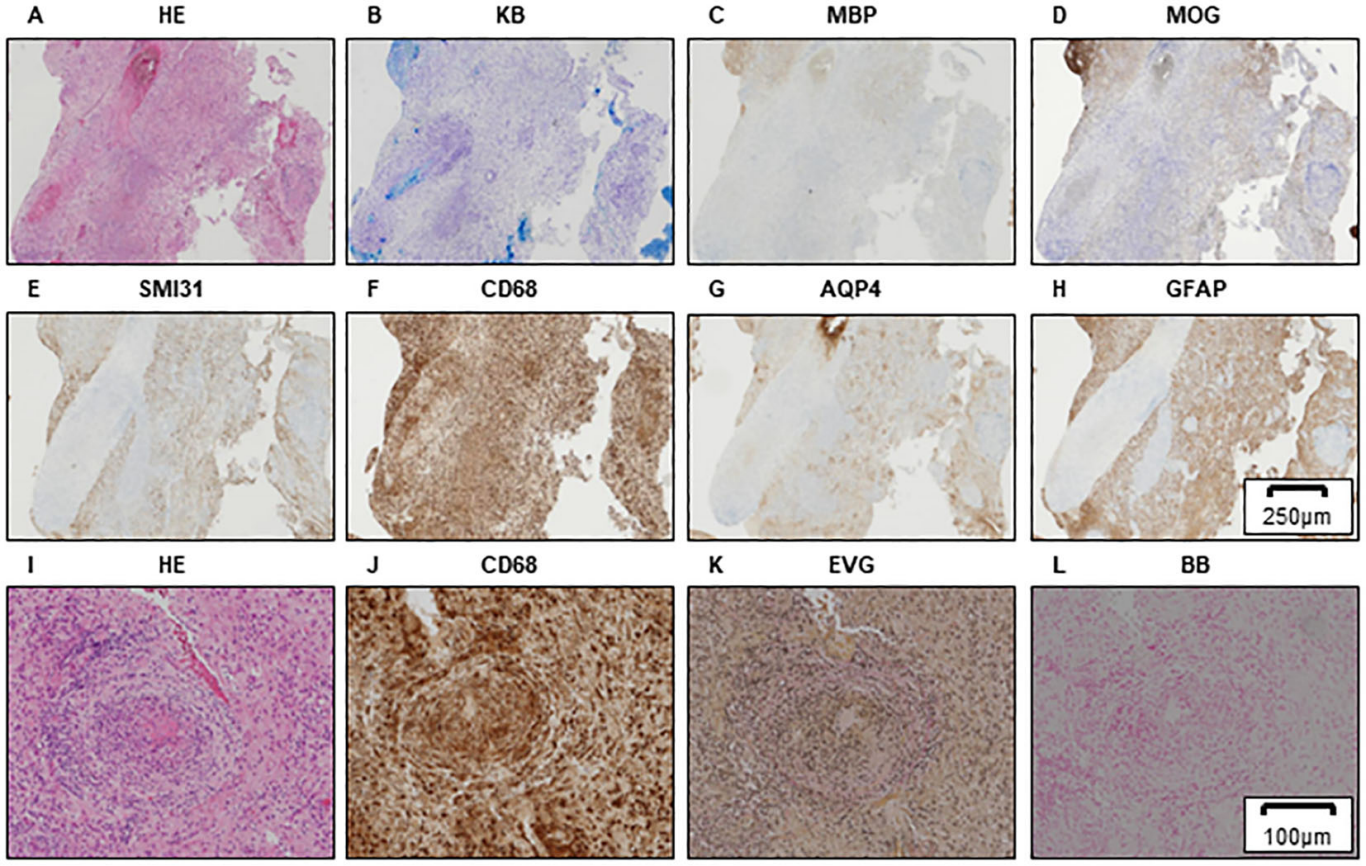
Brain biopsy of the left frontal lobe in Case 1. HE **(A)**, KB **(B)**, MBP **(C)**, and MOG **(D)** staining showing myelin destruction with inflammatory cell infiltration. A motor neuron marker (SMI31) staining demonstrating axonal damage in demyelinated lesions **(E)**. Inflammatory cells expressing CD68 **(F)**. Astrocyte loss can be observed to be relatively preserved by AQP4 **(G)** and GFAP **(H)** staining. Scale bar, 250 µm. Higher-magnification brain biopsy of the left frontal lobe in Case 1 is shown **(I–L)**. HE **(I)** and CD68 **(J)** staining showing the CD68-positive small round cell infiltrates around small blood vessels. Absence of Elastica van Gieson (EVG) staining reveals that vessels with CD68-positive cell infiltration are veins **(K)**. Berlin blue staining (BB), which demonstrates iron deposits, shows that hemorrhagic lesions are not detected around the vessels; therefore, the involvement of vasculitis is negative **(L)**. **(A–H)** Scale bar, 250 µm; **(I–L)** scale bar, 100 µm. HE, hematoxylin and eosin; KB, Klüver–Barrera staining; MBP, myelin basic protein; MOG, myelin oligodendrocyte glycoprotein; AQP4, aquaporin4; GFAP, glial fibrillary acidic protein; EVG, Elastica van Gieson; BB, Berlin blue staining.

Histopathological studies from previous hospitals showed reduced myelin sheath staining and myelin phagocytosis by Klüver–Barrera (KB) staining and highly reduced staining by myelin basic protein (MBP) and MOG immunostaining (**Figures 1A–D**). Severe axonal loss can be observed in the demyelinating lesion (**Figure 1E**). Small round cell infiltrates are observed around the small blood vessels (**Figure 1F**), and astrocytic damage is relatively mild (**Figures 1G, H**). Hemorrhagic lesions are not observed in areas with inflammatory cell infiltration (**Figures 1I–L**); thus, vasculitis was not involved. Moreover, demyelinating lesions were highly observed at these round cell infiltrating areas and these findings were known as “perivenous demyelination and inflammation,” which was characteristic in ADEM (**Figures 1A–F**, **I–K**).

**Figure 2:**
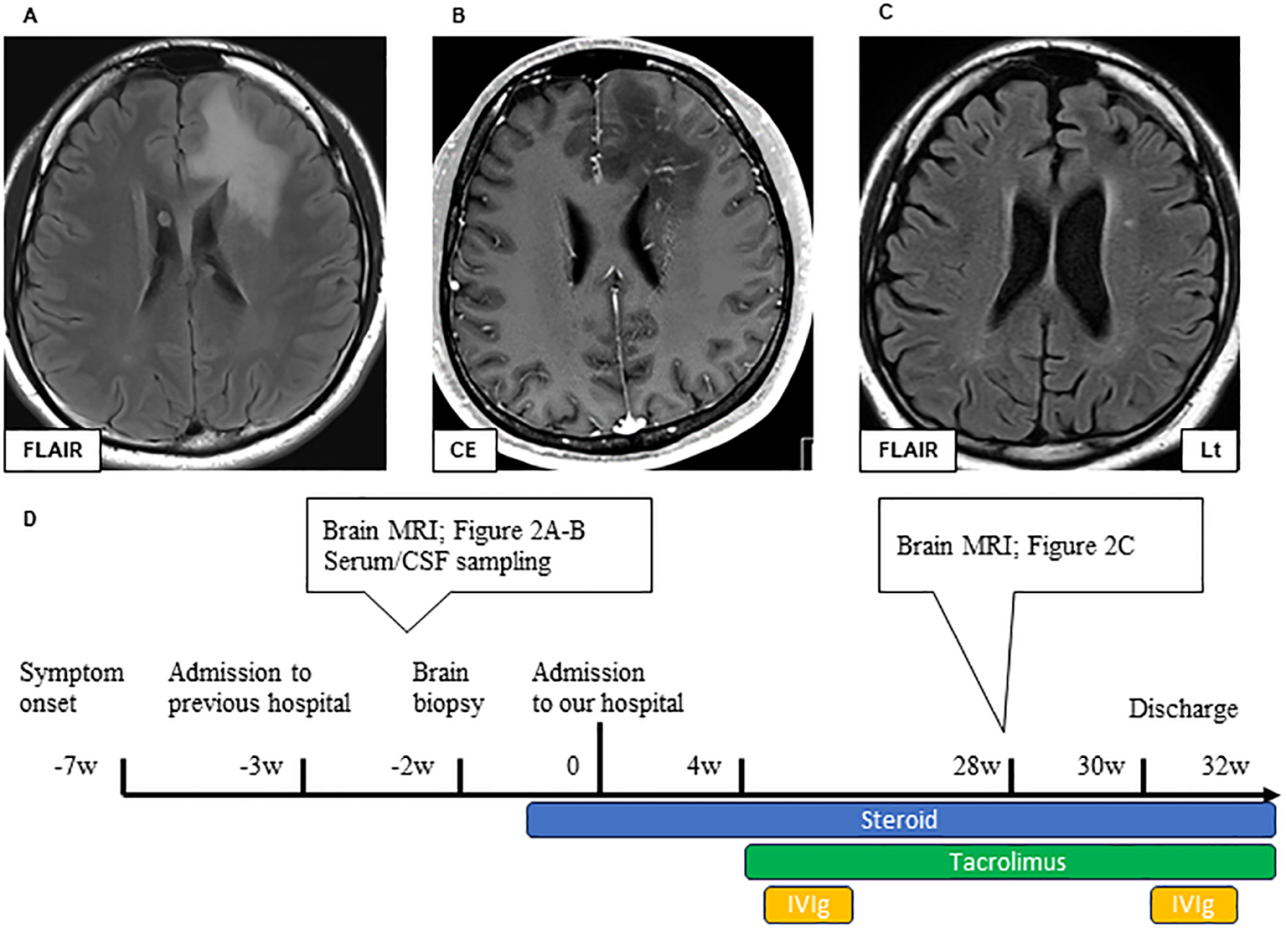
Brain MRI lesions in Case 1 during pre-treatment **(A, B)** and post-treatment **(C)**. Axial FLAIR **(A)** and CE T1 **(B)** sequences of brain MRI during pre-treatment. A large bilateral frontal FLAIR hyperintense lesion can be observed **(A)**. Extensive white matter lesions with linearly radiating gadolinium enhancement are observed around the ventricles. **(B)** Axial FLAIR of the brain MRI post-treatment **(C)**. Immunological treatment improved the FLAIR high-intensity lesions, but significant cerebral atrophy could be observed. The timeline scheme of MRI, brain biopsy, and immunological therapy of Case 1 **(D)**. FLAIR, fluid-attenuated inversion recovery; MRI, magnetic resonance imaging; CE, contrast enhancement; CSF, cerebrospinal fluid; IVIG, intravenous immunoglobulin therapy.

Before performing brain biopsy and initiating steroid therapy, the areas of white matter lesions showed linear-radiating gadolinium enhancement and high signal intensity on T2-weighted MRI (**Figures 2A, B**). Steroid therapy decreased the area of these lesions (**Figure 2C**), and the patient’s level of consciousness improved; however, severe cerebral atrophy persisted. Rehabilitation was performed and the patient became ambulatory (**Figure 2D**). After an 8-month hospitalization, the patient was discharged with a slightly higher cognitive dysfunction, such as the Mini-Mental State Examination (MMSE) [29 points (9 points for orientation, 5 points for calculation, 6 points for memory, and 9 points for language ability)] and Frontal Assessment Battery (FAB) [11 points (2 points for similarities, 2 points for fluency, 0 points for Luria’s motor series, 3 points for conflictual instructions, 1 point for Go–No-Go task, and 3 points for prehension behavior)].

## 3 Case 2

An 80-year-old woman with a history of bronchial asthma and hypertension was admitted to another hospital because of a rapid decline in consciousness over 1 week. At the time of admission to the previous hospital, the patient’s level of consciousness was E3V1M4 on the Glasgow Coma Scale, with minimal spontaneous speech and response to stimuli. In addition, severe limb paralysis was observed. MRI revealed disseminated white matter brain lesions with contrast enhancement (CE), and CSF analysis revealed an elevated cell count. A brain biopsy of the left occipital lobe was performed. Steroid therapy (dexamethasone 8 mg/day) resulted in a slight improvement in consciousness. After 3 months, she was transferred to our hospital for further examination and treatment. The level of consciousness at the time of admission to our hospital was E3V2M5 on the Glasgow Coma Scale, showing slight improvement in response to stimuli.

Serum tests and routine blood tests showed normal results. The CSF showed no abnormal change in nucleated cells (10/µL), protein (39 mg/dL), glucose (62 mg/dL), and IgG index (0.53). OCBs were not detected. Only anti-MOG antibody was positive (measured by qualitative cell-based assay); the other routine antibodies surveyed—AE-related antibodies against NAE, GFAP, N-methyl-D-aspartate receptor (NMDAR), leucine-rich glioma-inactivated 1 (LGI1), contactin-associated protein-like 2 (CASPR2), amino-3-hydroxy-5-methyl-4-isoxazolepropionic acid receptor (AMPAR), gamma-aminobutyric-acid B receptor (GABAbR), dipeptidyl-peptidase-like protein-6 (DPPX), glycine receptor (GlyR), glutamic acid decarboxylase 65 (GAD65), and PNS-related antibodies—were negative in the serum and/or CSF. These tests were conducted on samples obtained at the time of the admission to our hospital.

**Figure 3:**
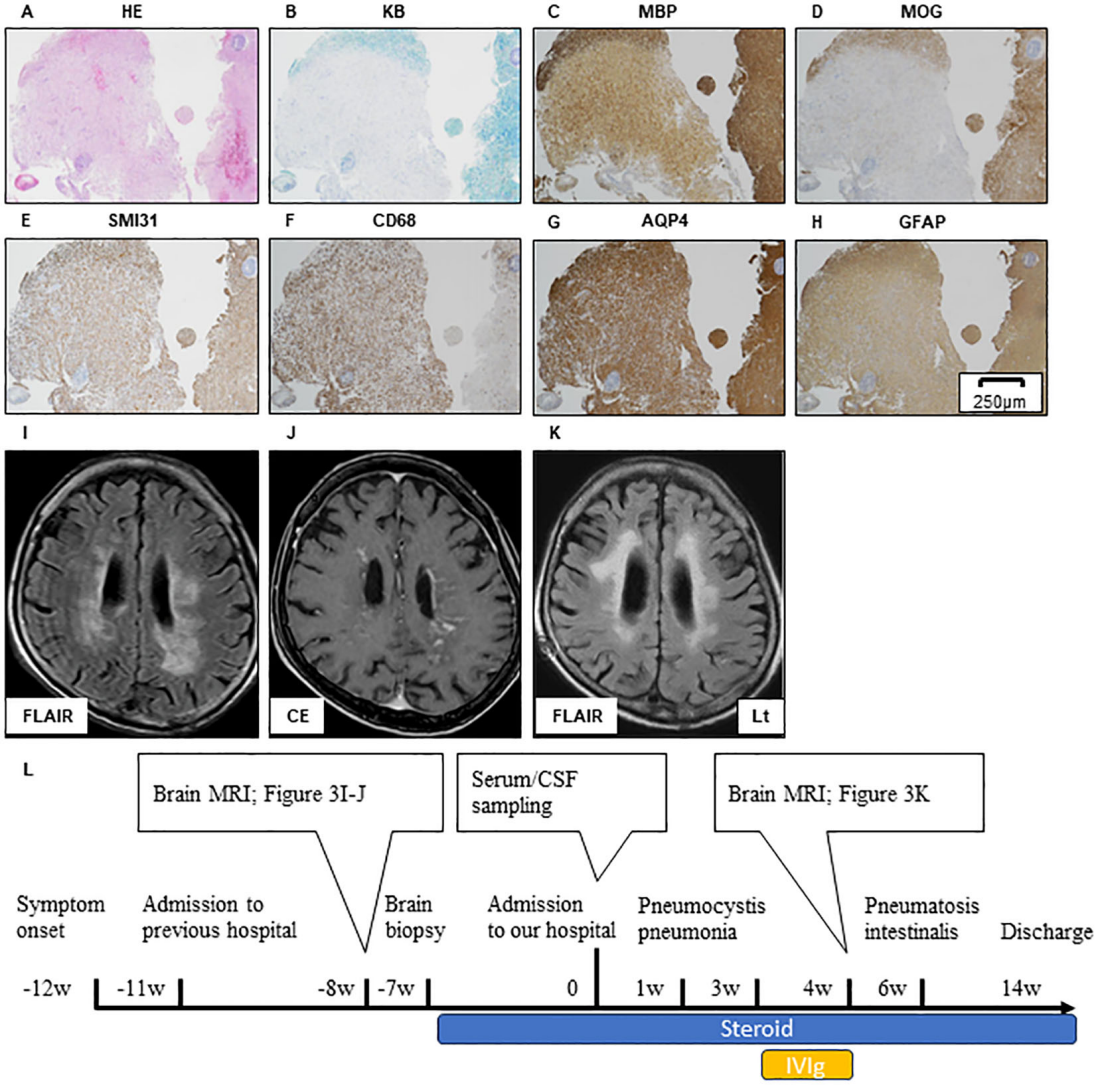
Brain biopsy of the left occipital lobe in Case 2. HE **(A)**, KB **(B)**, MBP **(C)**, and MOG **(D)** stain showing the myelin destruction with inflammatory cell infiltration. SMI31 stain reveals that the axonal damage in the demyelinated sight is relatively mild compared to that in Case 1 **(E)**. Inflammatory cell expressing CD68 **(F)**. Immunohistochemistry against AQP4 **(G)** and GFAP **(H)** demonstrates that the astrocyte damage is not clear. Scale bar, 250 µm. Brain MRI lesion in Case 2 during pre-treatment and post-treatment is shown **(I–K)**. Axial FLAIR **(I)** and CE T1 **(J)** sequence of brain MRI during pre-treatment. A large FLAIR-hyperintense lesion is located in bilateral deep white matter **(I)**. Widespread white matter lesions with a linear-radiating gadolinium enhancement can be observed **(J)**. Axial FLAIR of brain MRI during post-treatment. Immunological treatment had no effect on FLAIR high-intensity lesion progression, but the cerebral volume is relatively preserved **(K)**. The timeline scheme of MRI, brain biopsy, immunological therapy, and adverse events of Case 2 **(L)**. HE, hematoxylin and eosin; KB, Klüver–Barrera staining; MBP, myelin basic protein; MOG, myelin oligodendrocyte glycoprotein; AQP4, aquaporin4; GFAP, glial fibrillary acidic protein; FLAIR, fluid-attenuated inversion recovery; MRI, magnetic resonance imaging; CE, contrast enhancement; CSF, cerebrospinal fluid; IVIG, intravenous immunoglobulin therapy.

A brain biopsy performed at the previous hospital indicated myelin destruction and an ADEM-like lesion, similar to that in Case 1 (**Figures 3A–H**). In contrast to Case 1, SMI31 immunostaining did not show severe axonal loss within the demyelinated lesions, and only swollen axons can be observed (**Figure 3E**).

The EEG showed irregular slow waves with medium to high amplitudes in the right temporal lobe and no sharp waves. Moreover, the total body CT scan showed no evidence of tumor.

**Figure 4:**
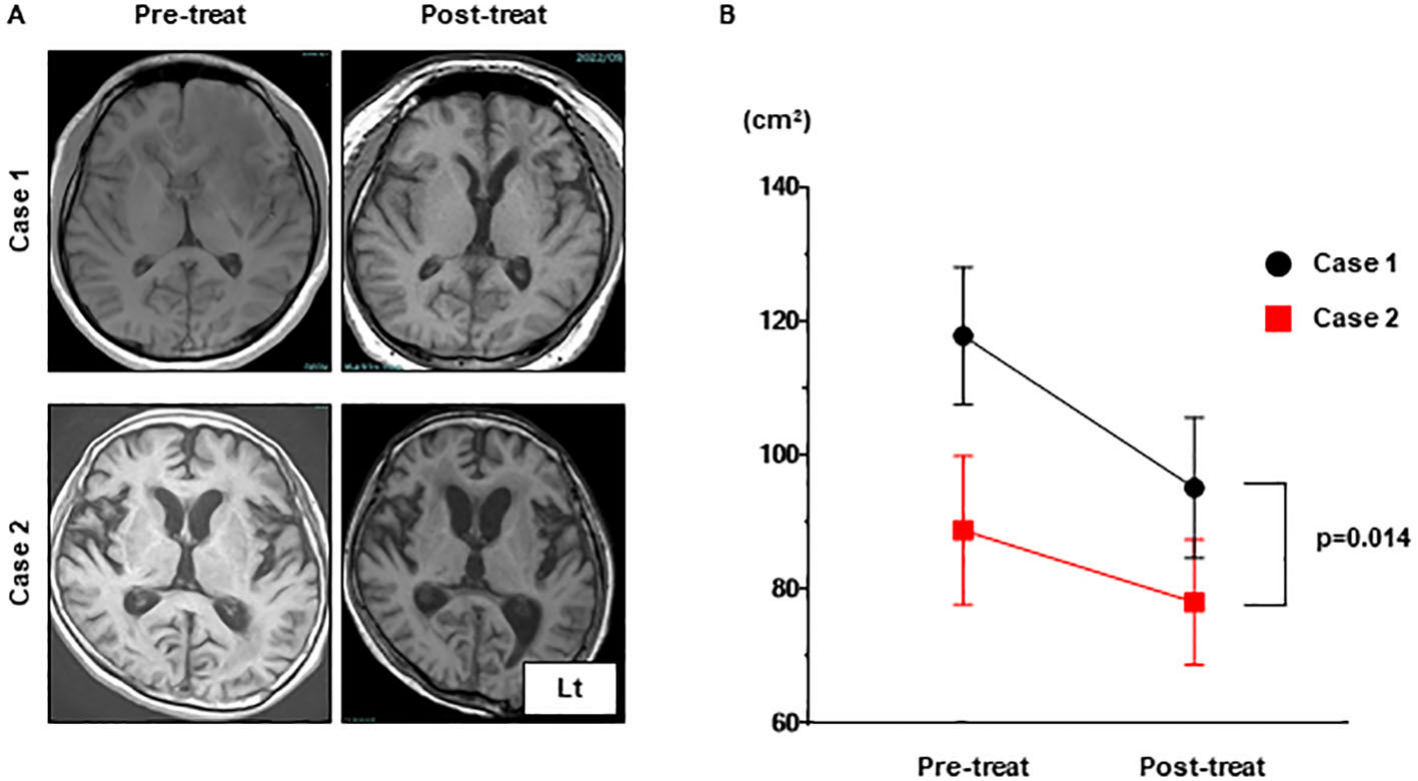
The progression of brain atrophy in Case 1 was significantly more advanced compared to that in Case 2 as can be observed in the MRI T1 images. **(A)** The brain volumes in MRI T1 sequences of Case 1 and Case 2 were compared pre-treatment and post-treatment. **(B)** The brain volumes measured using ImageJ for both were analyzed with a one-way analysis of variance (ANOVA). MRI, magnetic resonance imaging.

After admission, the patient developed pneumocystis pneumonia and received antibiotic therapy. Subsequently, intravenous immunoglobulin (IVIg) was administered; however, the patient’s symptoms did not improve. Six weeks after the admission, pneumatosis intestinalis was observed and immunological therapy was discontinued. Before treatment, MRI shows widespread white matter lesions with linear-radial gadolinium enhancement (**Figures 3I, J**). FLAIR high-intensity lesion on MRI after immunological treatment (**Figure 3K**) was not altered (**Figures 3I, K, L**), but the cerebral volume was relatively preserved compared to that in Case 1 (**Figures 4A, B**). The patient was transferred after 2 months of treatment. The level of consciousness at the time of discharge was E3V2M5 on the Glasgow Coma Scale and no improvement in the severe limb paralysis was observed.

## 4 Discussion

Herein, we present two cases of MOGAD exhibiting a distinctive linear-radiating gadolinium enhancement on MRI. Brain biopsies from both cases revealed pathological findings consistent with ADEM, characterized by perivenous demyelination and inflammation. Additionally, axonal damage was observed in Case 1, whereas it was not identified in Case 2, highlighting a difference between the two cases.

MOGAD was recently identified as a novel inflammatory central nervous system (CNS) demyelinating disease (1). The clinical spectrum of MOGAD overlaps with that of MS, ADEM, NMOSD, and cerebral cortical encephalitis (2). MOGAD is characterized by the presence of an antibody against MOG in the outer layers of the myelin sheath. Experimental autoimmune encephalomyelitis (EAE) is an animal model of inflammatory demyelinating disease characterized by the primary and secondary production of MOG-specific lymphocytes and MOG antibodies (6). Thus, MOG antibodies are considered to be pathogenic autoantibodies involved in human inflammatory demyelinating diseases.

MOGAD has been reported to exhibit diverse and specific imaging findings (4, 7). A previous report suggested that MRI characteristics of MOGAD can be classified into four types (4), namely, multifocal hazy/poorly marginated lesions, extensive and periventricular white matter lesions, cortical encephalitis with leptomeningeal enhancement/brain atrophy, and tumefactive demyelinating lesions (4). However, radial linear periventricular enhancement, which was observed in our patient, cannot be assigned to these categories. Radial linear periventricular enhancement has been reported in patients with autoimmune GFAP astrocytopathy, lymphomatoid granulomatosis, neurosarcoidosis, and CNS vasculitis (8–13), but these diseases were pathologically and serologically ruled out. Additionally, similar radiological patterns have been reported in cases of encephalitis with negative anti-GFAP antibodies (14, 15). In these reports, MOG antibodies were also negative. Although one case with a brain biopsy has been reported (15), it showed astrogliosis, which is compatible with the histology of GFAP astrocytopathy, unlike our findings. These observations suggest that various etiologies may underlie the MRI finding of linear radiating gadolinium enhancement.

Pathological features of MOGAD differ from those in multiple sclerosis and AQP4-IgG-positive NMOSD, suggesting that MOGAD is an independent clinical and pathological entity (16). Brain biopsy samples from our two cases revealed perivenous demyelination and inflammation, which have been reported as characteristic histology in both ADEM and MOGAD (17–19). This perivenous inflammation is possibly the cause of the linear radiating gadolinium enhancement observed on MRI. Consistent with this hypothesis, brain biopsies from GFAP astrocytopathy patients have shown inflammatory responses around perivascular regions (20), suggesting that the linear radiating gadolinium enhancement on MRI indicates common histology of the brain. Axonal damage observed only in Case 1 has rarely been reported in ADEM or MOGAD (17, 21). The difference in axonal damage between Case 1 and Case 2 may serve as a reason to explain the presence of severe brain atrophy in Case 1 compared with Case 2. These results suggest that pathological findings may predict neurological damage.

In conclusion, our study demonstrated that radial linear periventricular enhancement on MRI may be observed in patients with MOGAD. Moreover, brain biopsies of our cases not only revealed the absence of tumors, vasculitis, and astrocytopathy contributing to the pathology, but also suggested that the presence of axonal damage might serve as a prognostic marker for brain atrophy. Therefore, it is important to consider MOGAD as a differential diagnosis even in atypical imaging and, if necessary, to perform a brain biopsy for diagnosis and predicting the clinical prognosis.

## Data Availability

The original contributions presented in the study are included in the article/supplementary material. Further inquiries can be directed to the corresponding author.

## References

[1] Jurynczyk M Geraldes R Probert F Woodhall MR Waters P Tackley G. Distinct brain imaging characteristics of autoantibody-mediated CNS conditions and multiple sclerosis. Brain. (2017) 140:617–27. doi: 10.1093/brain/aww350 28364548

[2] Bennett JL Costello F Chen JJ Petzold A Biousse V Newman NJ. Diagnosis of myelin oligodendrocyte glycoprotein antibody-associated disease: International MOGAD Panel proposed criteria. Lancet Neurol. (2023) 22:89–100. doi: 10.1016/S1474-4422(22)00187-9 36706773

[3] Cortese R Battaglini M Prados F Bianchi A Haider L Jacob A. Clinical and MRI measures to identify non-acute MOG-antibody disease in adults. Brain. (2023) 146:2489–501. doi: 10.1093/brain/awac480 36515653

[4] Wang J Qiu Z Li D Yang X Ding Y Gao L. Clinical and imaging features of patients with encephalitic symptoms and myelin oligodendrocyte glycoprotein antibodies. Front Immunol. (2021) 12:722404. doi: 10.3389/fimmu.2021.722404 34691028 PMC8529193

[5] Fang B McKeon A Hinson SR Kryzer TJ Pittock SJ Aksamit AJ. Autoimmune glial fibrillary acidic protein astrocytopathy: A novel meningoencephalomyelitis. JAMA Neurol. (2016) 73:1297–307. doi: 10.1001/jamaneurol.2016.2549 27618707

[6] Reindl M Di Pauli F Rostásy K Berger T. The spectrum of MOG autoantibody-associated demyelinating diseases. Nat Rev Neurol. (2013) 9:455–61. doi: 10.1038/nrneurol.2013.118 23797245

[7] Salunkhe M Gupta P Singh RK Tayade K Goel V Agarwal A. Clinical and radiological spectrum of anti-myelin oligodendrocyte glycoprotein (MOG) antibody encephalitis: single-center observational study. Neurological Sci. (2023) 44:2475–89. doi: 10.1007/s10072-023-06686-z 36810716

[8] Flanagan EP Hinson SR Lennon VA Fang B Aksamit AJ Morris PP. Glial fibrillary acidic protein immunoglobulin G as biomarker of autoimmune astrocytopathy: Analysis of 102 patients. Ann Neurol. (2017) 81:298–309. doi: 10.1002/ana.24881 28120349

[9] Shoemaker EI Lin ZS Rae-Grant AD Little B. Primary angiitis of the central nervous system:unusual MR appearance. Am J Neuroradiol. (1994) 15:331–4. PMC8334615 8192081

[10] Williams DW 3rd Elster AD Kramer SI. Neurosarcoidosis: gadolinium-enhanced MR imaging. J Comput Assist Tomogr. (1990) 14:704–7. doi: 10.1097/00004728-199009000-00004 2398145

[11] Tateishi U Terae S Ogata A Sawamura Y Suzuki Y Abe S. MR imaging of the brain in lymphomatoid granulomatosis. Am J Neuroradiol. (2001) 22:1283–90. PMC7975211 11498415

[12] Ganta K Malik AM Wood JB Levin MC. Radial contrast enhancement on brain magnetic resonance imaging diagnostic of primary angiitis of the central nervous system: a case report and review of the literature. J Med Case Rep. (2014) 8:26. doi: 10.1186/1752-1947-8-26 24468474 PMC3917527

[13] Hassan AS Trobe JD McKeever PE Gebarski SS. Linear magnetic resonance enhancement and optic neuropathy in primary angiitis of the central nervous system. J Neuroophthalmol. (2003) 23:127–31. doi: 10.1097/00041327-200306000-00004 12782924

[14] Yue J Lin P Lian C Yao H Jiang L Liao S. Brain radial enhancement pattern in patients with negative glial fibrillary acidic protein-IgG: A cases series study. J Neurological Sci. (2023) 453:120782. doi: 10.1016/j.jns.2023.120782 37683309

[15] Wickel J Chung HY Kirchhof K Boeckler D Merkelbach S Kuzman P. Encephalitis with radial perivascular emphasis: Not necessarily associated with GFAP antibodies. Neurology(R) Neuroimmunology Neuroinflamm. (2020) 7:e670. doi: 10.1212/NXI.0000000000000670 PMC7051210 32019875

[16] Shu H Ding M Shang P Song J Lang Y Cui L. Myelin oligodendrocyte glycoprotein antibody associated cerebral cortical encephalitis: Case reports and review of literature. Front Hum Neurosci. (2022) 15:782490. doi: 10.3389/fnhum.2021.782490 35046784 PMC8762331

[17] Höftberger R Guo Y Flanagan EP Lopez-Chiriboga AS Endmayr V Hochmeister S. The pathology of central nervous system inflammatory demyelinating disease accompanying myelin oligodendrocyte glycoprotein autoantibody. Acta Neuropathologica. (2020) 139:875–92. doi: 10.1007/s00401-020-02132-y PMC7181560 32048003

[18] Misu T. Pathology of myelin oligodendrocyte glycoprotein antibody–associated disease. Clin Exp Neuroimmunol. (2020) 12:5–6. doi: 10.1111/cen3.12617

[19] Takai Y Misu T Kaneko K Chihara N Narikawa K Tsuchida S. Myelin oligodendrocyte glycoprotein antibody-associated disease: An immunopathological study. Brain. (2020) 143:1431–46. doi: 10.1093/brain/awaa102 32412053

[20] Long Y Liang J Xu H Huang Q Yang J Gao C. Autoimmune glial fibrillary acidic protein astrocytopathy in Chinese patients : a retrospective study. Eur J Neurol. (2018) 25:477–83. doi: 10.1111/ene.13531 29193473

[21] Pohl D Alper G Van Haren K Kornberg AJ Lucchinetti CF Tenembaum S. Acute disseminated encephalomyelitis: Updates on an inflammatory CNS syndrome. Neurology. (2016) 87:S38–45. doi: 10.1212/WNL.0000000000002825 27572859

